# Enzymatic Synthesis of Human Milk Fat Substitute - A Review on Technological Approaches

**DOI:** 10.17113/ftb.59.04.21.7205

**Published:** 2021-12

**Authors:** Hasrul Abdi Hasibuan, Azis Boing Sitanggang, Nuri Andarwulan, Purwiyatno Hariyadi

**Affiliations:** 1 Department of Food Science and Technology, Faculty of Agricultural Engineering and Technology, IPB University (Bogor Agricultural University), Campus IPB Dramaga, Raya Dramaga, 16680 Bogor, Indonesia; 2 Indonesian Oil Palm Research Institute (IOPRI), Brigjen Katamso 51, 20158 Medan, Indonesia; 3 Southeast Asian Food and Agricultural Science and Technology (SEAFAST) Center, IPB University (Bogor Agricultural University), Campus IPB Dramaga, Raya Dramaga, 16680 Bogor, Indonesia

**Keywords:** human milk fat substitute, interesterification, lipase, *sn*-2 palmitate

## Abstract

Human milk fat substitute (HMFS) is a structured lipid designed to resemble human milk fat. It contains 60-70% palmitic acid at the *sn*-2 position and unsaturated fatty acids at the *sn*-1,3 positions in triacylglycerol structures. HMFS is synthesized by the enzymatic interesterification of vegetable oils, animal fats or a blend of oils. The efficiency of HMFS synthesis can be enhanced through the selection of appropriate substrates, enzymes and reaction methods. This review focuses on the synthesis of HMFS by lipase-catalyzed interesterification and provides a detailed overview of biocatalysts, substrates, synthesis methods, factors influencing the synthesis and purification process for HMFS production. Major challenges and future research in the synthesis of HMFS are also discussed. This review can be used as an information for developing future strategies in producing HMFS.

## INTRODUCTION

Fat is a component of human milk that gives half of the energy required for babies (*1*-*3*). Human milk contains 3-5% fat comprising 98% triacylglycerols (TAGs) (*4*-*6*). The main fatty acids in the human milk TAGs are oleic (30-35%), palmitic (20-30%), linoleic (7-14%) and stearic (5.7-8%) acids (*5*). About 60-70% of palmitic acid is located at the *sn*-2 position, whereas unsaturated fatty acids (*i.e.* oleic, linoleic and ω-3 polyunsaturated fatty acids (PUFAs)) are at the *sn*-1,3 positions in the TAG structure of human milk fat (HMF) (*6*). Palmitic acid at the *sn*-2 position has a significant importance especially in the absorption and metabolism of lipids and nutrition of infants (*7*, *8*).

The dominant TAGs in HMF are 1,3-dioleoyl-2-palmitoyl-glycerol (OPO; 16-29%) and 1-oleoyl-2-palmitoyl-3-linoleoylglycerol (OPL; 13-20%) (*9*). TAGs with palmitic acid at *sn*-2 position are very helpful in effectively promoting the absorption of calcium ions, leading to the formation of softer stools and hence reducing the possibility of constipation (*1*, *6*, *10*, *11*). TAGs containing palmitic acid at *sn*-2 position can also increase early bone mineralization and growth, affect the composition of intestinal microflora, decrease the level and severity of intestinal inflammation and influence neurobiology, which includes modulation of early infant crying (*12*).

The composition and distribution of fatty acids in HMF are used as a basis to develop an alternative fat as an ingredient for infant formulas. As sources of nutrients, infant formulas are an alternative to human milk when nursing mother does not produce enough breast milk (*13*, *14*). Fats commonly used for infant formulas are vegetable oils or animal fats, especially bovine milk fat (*3*). However, the composition and distribution of fatty acids in vegetable oils and mammalian milk fats differ from that of HMF (*4*). In vegetable oils, palmitic acid is mainly (>80%) esterified to *sn*-1,3 positions (*9*). Meanwhile, animal fat such as cow’s milk fat has similar palmitic acid content as HMF, but the percentage of palmitic acid esterified at *sn*-2 position is only about 40% (*4*, *14*). Hereby, vegetable oils, animal fats or blends of oils are modified to mimic the composition and distribution of fatty acids found in HMF (*15*, *16*). This modified fat is so-called human milk fat substitute (HMFS) (*14*, *17*).

HMFS has been successfully developed and commercialized as energy supplement and considered as a source of essential fatty acids, and as nutritional supplement in infant formulas (*4*). Based on the categories of the LIPID MAPS® structure database (*18*), HMFSs are classified into four types based on their lipid structure, namely: (*i*) *sn*-2 palmitate (palmitic acid at the *sn*-2 position) such as OPO, (*ii*) long-chain polyunsaturated fatty acids, such as docosahexaenoic acid (DHA, 22:6 ω-3), eicosapentaenoic acid (EPA, 22:5 ω-3) and arachidonic acid (ARA, 20:4 ω-6), (*iii*) medium-chain triglycerides (MCTs) (having medium-chain fatty acids (MCFAs) with 6-12 carbon length), and (*iv*) milk fat globule membrane supplements (*4*).

Synthesis of HMFS is conducted by the enzymatic interesterification of oils and fats. The enzymatic interesterification operates at relatively low temperature, and it is considered cost-effective and environmentally friendly method (*19*). The interesterification utilizes lipase as a biocatalyst, which has specificity and selectivity to produce desired lipids with relatively low amount of by-products (*20*). Hereby, the changes in the structure of TAGs can be specifically regulated at *sn*-1,3, *sn*-2 or an unspecified position (*21*-*25*).

The development of structured lipids using enzymatic process technology has several challenges especially for tailoring higher catalytic efficiency and enzyme stability, which are important for overall productivity (*26*). HMFS contains more than 70% palmitic acid at *sn*-2 position and it can be produced by acidolysis in solvent system between tripalmitin and a mixture of hazelnut oil fatty acids and stearic acid using Lipozyme RM IM (*15*), or tripalmitin and fatty acids from hazelnut oil and γ-linolenic acid (GLA) using Lipozyme RM IM and Lipozyme TL IM (*16*). He *et al.* (*6*) reported that acidolysis of TAGs from *Nannochloropsis oculata* and fatty acids from *Isochrysis galbana* using Novozyme 435, Lipozyme TL IM, Lipozyme RM IM and recombinant *Candida antarctica* Lipase B (recombinant CAL-B) in solvent-free system produced HMFS containing 59.38–68.13% palmitic acid at *sn*-2 position.

The reported studies on HMFS production highlighted the exploration of the use of new oils and fats, finding more cost-effective catalysts, synthesis methods, reactor configurations and purification process. Wei *et al.* (*4*) reviewed the achievements and trends of development of HMFS, with focus on nutritional bases, preparation methods and applications of HMFS. As an addition to that comprehensive review, this work is more focused on the utilization of lipase as a biocatalyst and factors that affect lipase-catalyzed synthesis of HMFS. It starts with the biocatalysts used for production of HMFS, followed by substrates, methods and reactor configurations, factors influencing the synthesis and purification of HMFS, with a specific objective to increase the efficiency of HMFS synthesis. Additionally, the developments of HMFS production including challenges and opportunities for future research of HMFS are also presented in this work.

## LIPASE FOR HMFS SYNTHESIS

Lipase (triacylglycerol acyl-hydrolase, EC 3.1.1.3) is commonly used for oil or fat hydrolysis. In non-aqueous media, lipase can also catalyze the esterification, acidolysis, alcoholysis and interesterification (*20*, *27*-*29*). The lipase-catalyzed interesterification involves the reversible reaction of simultaneous hydrolysis and esterification reactions (*30*). A small amount of water is important for non-aqueous enzymatic catalysis for maintaining active conformational structure of the enzymes during non-covalent interactions (*31*). The excess water has to be removed to shift the progress of the reaction from hydrolysis to esterification, thus enhancing the reaction yield. When the hydrolysis prevails over esterification, by-products such as glycerol, free fatty acids (FFA), monoacylglycerol (MAG) and diacylglycerol (DAG) are obtained, which eventually hampers the separation process.

As part of non-aqueous reaction, the esterification of HMFS can be carried out by lipase as the biocatalyst. The sources of lipase are mostly microorganisms. The commercial lipases available on the market and mostly studied in recent years for the production of HMFS are derived from *Rhizomucor miehei*, *Thermomyces lanuginosa*, *Candida antarctica*, *Candida parapsilosis,* recombinant lipase B from *Candida antarctica*, *Candida lipolytica, Candida* sp. 99-125, *Rhizopus oryzae*, *Alcaligenes* sp. and *Mucor miehei* (*9*, *24*).

Lipases with regiospecificity and regioselectivity are of interest as the reaction yield can be tuned up by these properties. Additionally, the use of an immobilized lipase with a biocatalytic activity maintained at an industrial scale is required for multiple uses, ensuring the economic viability of the process (*32*, *33*), and thus lowering production costs (*34*, *35*). The immobilized lipase sometimes has a higher stability than the native one from freely suspended enzyme (*36*).

### Selectivity and/or specificity of lipases as biocatalysts for HMFS synthesis

Compared to chemical catalysts, lipases have functional properties: (*i*) substrate specificity, *i.e.* the ability to hydrolyse preferentially a type of acylglycerol, (*ii*) fatty acid specificity or typoselectivity, *i.e.* the ability to target a certain fatty acid or group of fatty acids, (*iii*) positional specificity or regioselectivity, *i.e.* the ability to distinguish the two external positions of the TAG glycerol backbone, and (*iv*) stereospecificity, *i.e.* the ability to distinguish between *sn*-1 and *sn*-3 positions of TAG molecule (*27*). The incorporation of fatty acids into a TAG structure is influenced by many factors, including the geometry of the binding sites of the lipases, free energy changes between the substrate and products, variation of pH values, effect of the chain length of fatty acids on the solubility of water and the physical state (*24*).

The most commonly commercialized lipases used for HMFS synthesis are Lipozyme RM IM (from *Rhizomucor miehei*), Lipozyme TL IM (from *Thermomyces lanuginosa*), and Novozyme 435 (from *Candida antarctica*). The three enzymes are immobilized with support materials such as ion-exchange resin, silica gel, and microporous anionic resin, respectively (*9*). These enzymes are specific for the interesterification of oils and fats. Lipozyme RM IM and Lipozyme TL IM are enzymes that show high regiospecificity at the *sn*-1,3 positions (*24*), while Novozyme 435 does not show positional specificity (*24*, *37*, *38*).

Generally, Lipozyme RM IM is used in acidolysis (*15*, *16*, *39*-*53*), although several studies also use it in transesterification reactions (*43*, *54*, *55*). Lipozyme TL IM is usually used in transesterification reaction (*56*-*59*), but some reported works also use it in acidolysis (*6*, *16*, *60*-*62*). Rodrigues and Fernandez-Lafuente (*20*) revealed that Lipozyme RM IM showed a higher activity in acidolysis, whereas Lipozyme TL IM in alcoholysis or transesterification. This can be due to the fact that Lipozyme RM IM is classified as an esterase and it is more specific for TAGs containing low-molecular-mass fatty acids at pH=5.3 than at pH=8.0 (*20*), while Lipozyme TL IM is quite stable with the maximum activity at a pH around 9 (*63*).

Tecelão *et al.* (*60*) reported that acidolysis of tripalmitin and oleic acid or PUFAs by Lipozyme RM IM produced HMFS containing 79.9% palmitic acid at the *sn*-2 position, higher than Lipozyme TL IM (75.6%). Lipozyme RM IM (68.13%) is also superior to Lipozyme TL IM (59.38%) in HMFS synthesis containing high palmitic acid at *sn*-2 position by acidolysis of microalgal oil from *Nannochloropsis oculate* and *Isochrysis galbana* (*6*). Lee *et al.* (*59*) reported that Lipozyme TL IM was used for interesterification between palm stearin and ethyl oleate at a molar ratio 1:5.5, at 50 °C for 3 h. The produced HMFS had 31.43% OPO content and 80.6% palmitic acid at *sn*-2 position. Karabulut *et al.* (*58*) produced HMFS containing 23.0% palmitic acid and 41.5% palmitic acid at *sn*-2 position of TAGs by transesterification among palm oil, palm kernel oil, olive oil, sunflower oil and marine oil at a mass ratio 4.0:3.5:1.0:1.5:0.2, at 60 °C for 6 h using Lipozyme TL IM.

Novozyme 435 is mostly used for HMFS synthesis in the interesterification of oils and fats that improves palmitic acid content at the *sn*-2 position with donors such as palmitic acid, ethyl palmitate or palm oil fractions. Generally, palm oil fractions have high palmitic acid content distributed at the *sn*-1,3 positions (*64*). The incorporation of fatty acids by acidolysis or transesterification using Novozyme 435 is affected by substrates. Novozyme 435 is a highly versatile catalyst that catalyzes a wide variety of different substrates due to its high enantioselectivity (*60*). Robles *et al.* (*65*) used Novozyme 435 for acidolysis of tuna fish oil and palmitic acid, and the produced TAG contained amount of substance fraction *x*(palmitic acid)=57% and 17% DHA at *sn*-2 position. Turan *et al.* (*66*) also used Novozyme 435 in acidolysis and transesterification reactions between hazelnut oil and palmitic acid or ethyl palmitate in a solvent-free system. The optimum conditions were hazelnut/ethyl palmitate at a molar ratio 1:6, temperature 65 °C and reaction time 17 h. Hereby, HMFS with *x*(palmitic acid)=48.6% and 35.5% palmitic acid at *sn*-2 position was obtained. Novozyme 435 is used in acidolysis of palm oil and a mixture of DHA and ARA to produce HMFS with 17.20% DHA+ARA incorporated at *sn*-2 position (*67*). Acidolysis of palm olein and a mixture of DHA, GLA and palmitic acid using Novozyme 435 produced HMFS with 35.11% palmitic acid at the *sn*-2 position (*68*). Novozyme 435 is also used in transesterification of a mixture of palm stearin, palm kernel oil, soybean oil, olive oil and tuna fish oil to produce HMFS with fatty acid composition resembling HMF (*69*).

### Reusability of lipase

Enzymes are immobilized to prevent denaturation and leakage so that the number of batches or the duration of synthesis can be increased. Enzyme is immobilized through adsorption, entrapment, covalent coupling or cross-linking (*36*). The enzyme immobilization yields (*i.e.* loading and recovered activity) strongly depend on the properties of the solid support such as the surface area, the number of accessible sides for binding, porosity and pore size (*33*). In addition, the hydrophilicity of the enzyme support is a factor that affects the reaction performance and the hydrophilicity of the support could be a beneficial side-effect of the immobilization (*70*).

Reusability of an immobilized lipase is very important issue to evaluate the operational stability (*6*, *60*); it is a major factor in determining the suitability of its utilization in different industries (*71*). Table 1 (*6*, *32*, *33*, *37*, *39*, *40*, *60*, *61*, *71*) shows the reusability of lipases for HMFS synthesis. It depends on the immobilization technique, inherent thermal properties of enzyme, reaction temperature and operational time. A gradual decrease of enzyme activity may be observed after several reaction batches. This is due to the denaturation (*72*) and/or loss of lipase immobilized during the reaction (*71*). In addition, the loss of enzyme activity may be due to a progressive dehydration occurring during the reaction (*33*). The multiple uses of the immobilized lipases can be expected due to the construction of the support that can protect the enzymes from mechanical inactivation and simultaneously inhibit lipase leakage (*73*).

**Table 1:** Reusability of lipase for human milk fat substitute synthesis

Lipase	Enzyme reusability	Reaction condition	Reference
Lipozyme RM IM	23 batches (1 batch for 1 h)	lard and soybean fatty acids (*r*=1:2.4), lipase loading 13.7%, temperature 61 °C, reaction time 1 h	(*39*)
Lipozyme RM IM	18 batch (1 batch for 2.5 h) using microfluidic packed reactor	tripalmitin and PUFA from microalgal oil (*r*=1:7), lipase loading 90 mg, temperature 60 °C	(*40*)
Lipozyme RM IM, Lipozyme TL IM, Novozyme 435 and *Candida parapsilosis*	system I: Lipozyme RM IM of 10 batches (230 h), half-life times for Lipozyme TL IM for 154 h (6.7 batch), Novozyme 435 for 253 h (11 batches) and *C. parapsilosis* for 276 h (12 batches) system II: half-life times of Novozyme 435 for 322 h (14 batches) and *C. parapsilosis* for 127 h (5.5 batches)	system I: tripalmitin and oleic acid (*r*=1:2), *m*(lipase)/*m*(tripalmitin)=8.9%, temperature 60 °C system II: tripalmitin and ω-3 PUFA (*r*=1:2), *m*(lipase)/*m*(tripalmitin)=8.9%, temperature 60 °C	(*60*)
Novozyme 435, recombinant CAL-B, Lipozyme TL IM and Lipozyme RM IM	20 batches (Novozyme 435, recombinant CAL-B, Lipozyme RM IM), 4 batches (Lipozyme TL IM) (1 batch for 24 h)	TAG from *Nannochloropsis oculata* and *Isochrysis galbana* fatty acids (*r*=1:3), lipase loading 10%, temperature 60 °C (Novozyme 435, Lipozyme TL IM), 50 °C (recombinant CAL-B, Lipozyme RM IM)	(*6*)
Novozyme 435	10 batches (1 batch for 24 h)	palm stearin and palmitic acid (*r*=1:3), lipase loading 10%, temperature 38 °C, solvent system	(*37*)
*Candida lipolytica* immbolized in magnetic multi-walled carbon nanotubes	20 batches (1 batch for 2 h)	tripalmitin and oleic acid (*r*=1:6), lipase loading 20 g/L, temperature 50 °C	(*71*)
*Rhizopus oryzae*	10 batches (1 batch for 19 h)	palm stearin containing high palmitic acid at *sn*-2 position and oleic acid (*r*=1:6), lipase loading 3.3%, temperature 50 °C	(*61*)
*Candida* sp. 99-125	5 batches (with additive β-cyclodextrin) (1 batch for 16 h)	lard and oleic acid (*m*_A_/*m*_B_=1:2), lipase loading 10%, temperature 45 °C	(*32*)
*Rhizopus oryzae* immobilized in Accurel® MP 1000 and Lewatit® VP OC 1600	half-life times for *Rhizopus oryzae* immobilized in Accurel® MP 1000 and in Lewatit® VP OC 1600 for of 34.5 and 64.0 h, respectively	tripalmitin and oleic acid (*r*=1:2), lipase loading 5%, temperature 60 °C, in solvent-free system	(*33*)

Zheng *et al.* (*71*) mentioned that *Candida lipolytica* immobilized in magnetic multi-walled carbon nanotubes (CLL@mMWCNTs) had a better activity and stability than Lipozyme RM IM and Lipozyme TL IM for interesterification between tripalmitin and oleic acid. Reusability of CLL@mMWCNTs was higher than that of Lipozyme RM IM, which was proven by 1.5-fold higher OPO content than with Lipozyme RM IM when reused for 20 cycles (1 cycle lasted 2 h). Immobilization of *C. lipolytica* on mMWCNTs *via* hydrophobic and cation-exchange interactions prevented the extensive conformational changes due to typical thermal denaturation (*71*). Tecelão *et al.* (*33*) reported that the best performance of *Rhizopus oryzae* lipase immobilized on Accurel® MP 1000 or Lewatit® VP OC 1600 was about 4-fold higher than on Eupergit® C regarding oleic acid incorporation in tripalmitin. *Rhizopus oryzae* lipase is immobilized on Accurel® MP 1000 and Lewatit® VP OC 1600 by physical adsorption. After the immobilization, glutaraldehyde is added to promote a stable crosslink between the lipase and the matrix, as well as to promote intermolecular bonds between the enzyme molecules. The immobilization of *R. oryzae* lipase on Eupergit® C can also be performed through direct enzyme binding on support *via* oxirane groups. However, enzymes immobilized on Eupergit® C through their different groups (amino, sulfhydryl, hydroxyl or phenolic) can block the substrate access to the enzyme active site, or can even lead to enzyme denaturation (*33*). In conclusion, as reported by Idris and Bukhari (*74*), materials and techniques for immobilization affect the conformational structure of enzymes related to catalytic properties.

## SOURCES AND TYPES OF SUBSTRATES FOR HMFS SYNTHESIS

Oils, fats or single TAG molecules can be used as substrates for HMFS synthesis (Table 2) (*6*, *40*, *45*, *48*-*52*, *65*, *66*, *68*, *75*-*80*). Several reported sources of palmitic acid for HMFS synthesis are tripalmitin (*77*), lard (*48*), palm stearin (*52*, *75*), basa catfish oil and its fraction (*51*), oil from *Nannochloropsis oculata* (*6*), palm oil (*78*) and palm olein (*68*). Oils that can be used as sources of oleic acids are high oleic sunflower oil (*79*), hazelnut oil (*66*), tea seed oil (*48*), rapeseed oil (*49*) and olive oil (*76*). Sunflower and soybean oil can be sources of linoleic acid (*45*). Flaxseed oil (*45*) and camelina oil (*77*) can be used as linolenic acid sources. Oils such as coconut (*45*) and palm kernel oil (*49*) can be used as MCFAs. Sources of EPA and DHA are fish oil (*65*, *80*), algal oil (*49*), microalgal oil from *Schizochytrium* sp. (*40*) and DHA single cell oil (*76*). Microbial oil is one of the sources of arachidonic acid (*49*). In addition, substrates that can be used as a single fatty acid donor for the synthesis of HMFS are palmitic or ethyl palmitic acid (*66*) as palmitic acyl donors, oleic acid (*75*) or ethyl oleate (*59*) as oleic acyl donors, linoleic acid (*75*), GLA (*16*), EPA and DHA (*46*) or ARA (*67*) as PUFA donors, and myristic (*52*), caprylic and capric acids (*41*) as acyl MCFA donors.

**Table 2:** Potential substrates for human milk fat substitute synthesis

Source	*w*(fatty acid)/%																	Reference
Total									*sn*-2 position							
C12:0	C16:0	C18:1	C18:2	C18:3	C20:4	C22:5	C22:6		C12:0	C16:0	C18:1	C18:2	C18:3	C20:4	C22:5	C22:6
Source of palmitic acid																		
Tripalmitin	-	94.2	1.7	1.0	-	-	-	-		na	na	na	na	na	na	na	na	(*77*)
Lard	0.7	28.5	35.9	9.4	-	-	-	-		-	78.9	10.0	3.7	-	-	-	-	(*48*)
Palm stearin	0.7	70.1	18.7	-	-	-	-	-		0.1	56.8	30.9	8.3	-	-	-	-	(*52*)
Fractionated palm stearin	-	91.6	2.3	0.4	-	-	-	-		-	92.0	3.7	0.6	-	-	-	-	(*75*)
*N. oculata* oil	-	37.9	10.6	2.1	0.7	-	-	-		-	76.2	2.0	1.1	0.4	-	-	-	(*6*)
Basa catfish oil	0.3	32.8	38.9	9.7	0.4	-	0.1	0.1		0.4	49.3	26.3	9.2	0.5	-	0.1	0.1	(*51*)
Solid fraction of basa catfish oil	0.3	34.6	37.3	9.2	0.4	-	0.1	0.1		0.5	60.4	18.8	8.6	0.4	-	0.1	0.1	(*50*)
Palm oil	-	53.6	34.1	8.7	-	-	-	-		-	18.9	62.3	18.5	-	-	-	-	(*78*)
Palm olein	-	43.6	40.9	9.9		-				-	13.8	66.4	19.0	-	-	-	-	(*68*)
Source of oleic acid																		
High oleic sunflower oil	-	7.1	74.1	9.6	1.7	-	-	-		-	2.8	38.3	43.8	-	-	-	-	(*79*)
Hazelnut oil	-	5.7	82.3	8.5	0.1	-	-	-		-	0.8	86.0	11.7	-	-	-	-	(*66*)
Tea seed oil	-	8.3	76.5	9.0	1.1	-	-	-		-	2.0	83.3	11.2	0.6	-	-	-	(*48*)
Rapeseed oil	0.9	6.7	58.1	22.5	7.8	-	-	-		-	1.1	54.6	33.9	0.5	-	-	-	(*49*)
Extra virgin olive oil	-	16.1	68.3	9.7	1.0	-	-	-		na	na	na	na	na	na	na	na	(*76*)
Source of linoleic acid																		
Sunflower oil	-	5.7	21.6	65.3	0.1	-	-	-		-	1.0	19.8	78.8	-	-	-	-	(*45*)
Soybean oil	-	10.6	23.4	53.2	5.8	-	-	-		-	1.2	22.9	68.3	5.4	-	-	-	(*45*)
Source of linolenic acid																		
Flaxseed oil	0.0	5.1	18.6	16.4	55.0	-	-	-		-	1.2	22.1	22.6	52.9	-	-	-	(*45*)
Camelina oil	-	6.3	18.1	19.4	35.5	-	-	-		na	na	na	na	na	na	na	na	(*77*)
Source of MCFA																		
Coconut oil	47.1	9.2	7.2	1.9	-	-	-	-		77.6	1.4	5.8	2.1	-	-	-	-	(*45*)
Palm kernel oil	55.7	6.4	10.2	2.1	-	-	-	-		42.3	8.7	25.2	5.4	-	-			(*49*)
Source of EPA and DHA																		
Fish oil	-	19.0	14.3	-	-	-	5.5	50.0		-	5.4	8.4	-	-	-	2.2	82.3	(*80*)
Tuna fish oil	-	22.0	18.7	2.3	-	-	0.6	18.4		-	20.5	2.6	-	-	-	1.5	33.5	(*65*)
Algal oil	-	24.7	1.4	0.4	0.3	-	15.4	42.0		-	14.6	2.4	2.2	0.3	-	13.7	47.8	(*49*)
Microalgal oil (*Schizochytrium* sp.)	-	19.1	16.2	4.1	-	-	16.1	38.6		-	8.3	38.7	1.9	-	-	11.5	34.0	(*40*)
DHA single cell oil	4.5	9.9	22.2	1.0	-	-	-	44.1		na	na	na	na	na	na	na	na	(*76*)
Source of arachidonic acid																		
Microbial oil	-	10.9	8.6	4.0	2.8	49.0	-	-		-	4.1	13.6	12.8	5.9	45.9	-	-	(*49*)

MCFA=medium-chain fatty acid, C12:0=lauric acid, C16:0=palmitic acid, C18:1=oleic acid, C18:2=linoleic acid, C18:3=linolenic acid, C20:4=arachidonic acid, C22:5=eicosapentaenoic acid (EPA), C22:6=docosahexaenoic acid (DHA), na=no analysis

The type of substrate is one of the important factors in the synthesis of HMFS. The composition of the raw material of the substrate that undergoes the interesterification process in the synthesis of HMFS has a significant influence on the final product. In the synthesis of HMFS with high content of palmitic acid at *sn*-2 position, it is better to use a substrate containing high content of palmitic acid at that position. Zhang *et al*. (*32*) produced HMFS containing 79.51% palmitic acid at the *sn*-2 position by acidolysis between lard (81.92% palmitic acid at the *sn*-2 position) and oleic acid (*r*=1:2) at 45 °C for 10 h using 10% additives β-cyclodextrin and *Candida* sp. 99-125. Yang *et al*. (*39*) also reported the production of HMFS with 71.1% palmitic acid at the *sn*-2 position obtained from the acidolysis between lard (67.3% palmitic acid at the *sn*-2 position) and fatty acids from soybean oil (*r*=1:2.4) at 61 °C for 1 h using 13.7% Lipozyme RM IM. In another study, Zou *et al*. (*50*) produced HMFS containing 58.43% palmitic acid at the *sn*-2 position by acidolysis between the solid fraction of basa catfish oil (60.42% palmitic acid at the *sn*-2 position) and fatty acids from high oleic acid sunflower oil (*r*=1:6) using 12% Lipozyme RM IM at 50 °C for 2 h. The use of basa catfish oil (49.3% palmitic acid at the *sn*-2 position), which was acidolyzed with fatty acids from sesame oil (*r*=1:3), using 8% Lipozyme RM IM at 40 °C for 2 h, yielded HMFS containing 48.3% palmitic acid at the *sn*-2 position (*51*).

The use of palm stearin containing different amounts of palmitic acid also affects the final quality of the product. Zou *et al*. (*52*) produced HMFS containing 62.8% palmitic acid at the *sn*-2 position from acidolysis between palm stearin (56.8% palmitic acid at the *sn*-2 position) and a mixture of fatty acids from rapeseed oil, sunflower oil, palm kernel oil, stearic acid and myristic acid (*r*=1:14.6) at 57 °C for 3.4 h using 10.7% Lipozyme RM IM. Meanwhile, Wang *et al*. (*75*) reported the use of fractionated palm stearin containing >90% tripalmitin (91.96% palmitic acid at the *sn*-2 position), which was acidolyzed with a combination of oleic acid and linoleic acid at *r*=1:8:4 and 60 °C for 4 h using 8% lipase NS 40086 yielding HMFS with 87.75% palmitic acid at the *sn*-2 position. In another study, Faustino *et al*. (*77*) acidolyzed tripalmitin (purity >85%) with fatty acids from camelina oil (*r*=1:2) at 60 °C for 24 h using *R. oryzae* lipase immobilized on 5% Lewatit VP OC 1600 yielding HMFS with 67.7% palmitic acid at the *sn*-2 position.

Melting points of some substrates for HMFS synthesis are 66-68 °C for tripalmitin (*77*), 30.3-48 °C for lard (*45*, *81*), 58-61 °C for palm stearin (*52*, *56*, *79*), 64.5 °C for fractionated palm stearin (*59*) and 63 °C for palmitic acid (*37*). The substrate melting point influences the enzymatic interesterification to obtain the optimal target product. Lee *et al*. (*81*) reported that transesterification between lard (27.1% palmitic acid) and olive oil (73.3% oleic acid) or camellia oil (81.6% oleic acid) at 40 °C for 12 h using 8.33% Lipozyme IM-20 in isooctane solvent yielded HMFS with 12.9 or 15.4% OPO, respectively. Transesterification of palm oil (44.3% palmitic acid) with olive oil or camellia oil resulted in HMFS containing 21.8 or 25.2% OPO. Despite having a high palmitic acid content at the *sn*-2 position, interesterification of lard produced lower OPO content than palm oil. This is related to the used low reaction temperature (40 °C), lower than the melting point of lard, which is 48 °C, therefore the solubility of lard is low in isooctane at 40 °C (*81*).

## METHODS AND REACTORS FOR LIPASE-CATALYZED HMFS SYNTHESIS

**Fig. 1:**
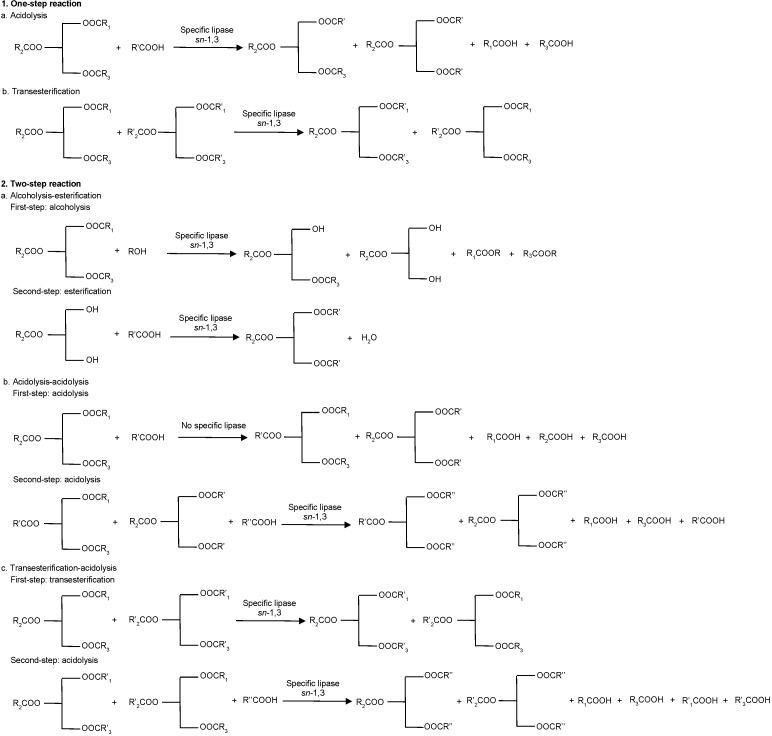
Schemes of lipase-catalyzed reactions for human milk fat substitute synthesis

Synthesis of HMFS can be carried out either through one-step reaction (*i.e.* acidolysis or transesterification) or two-step reaction (*i.e.* alcoholysis and esterification, acidolysis and acidolysis, or transesterification and acidolysis). The lipase-catalyzed reaction schemes for the synthesis of HMFS are shown in Fig. 1.

Acidolysis is a reaction between TAG and FFA (*4*). Enzymatic acidolysis is usually considered as a reversible two-step reaction, *i.e.* hydrolysis of the ester bonds with the released fatty acid followed by esterification of FFA to the backbone of glycerol (*5*). Table 3 (*6*, *15*, *16*, *32*, *39*, *40*, *44*-*53*, *60*, *62*, *67*-*69*, *75*, *76*, *81*) shows the reacting conditions for the HMFS production, where the resulting HMFS generally contains more than 60% palmitic acid at the *sn*-2 position. The main by-product of the acidolysis reaction are FFAs, which can be removed through neutralization (*65*, *82*), liquid-liquid extraction (*83*) or molecular distillation (*84*, *85*). In acidolysis, there is usually acyl migration in which fatty acids at the *sn*-2 position migrate to either *sn*-1 or *sn-*3 position (*9*). This entails the formation of various mixed TAGs as by-products in the reacting mixture. In general, acyl migration increases the separation complexity of the target HMFS from other by-products (*24*). Hence, the recovery of structured TAGs (*i.e.* target HMFS) is relatively lower than of those obtained by the two-stage process (*9*).

**Table 3:** Process conditions for the production of human milk fat substitute through acidolysis

Lipase	Substrate	Solvent system	Operation mode	Enzyme loading/%	*r(s*ubstrate)	Temperature/°C	Time/h	Product	Reference
Lipozyme RM IM	lard and soybean oil fatty acids	solvent-free system	batch	13.7	1:2.4	61	1	*w*(palmitic acid at *sn*-2 position)=71.1%	(*39*)
Lipozyme RM IM	tripalmitin and a mixture of hazelnut oil fatty acids and stearic acid	hexane	batch	10	1:12:1.5 (incorporation of oleic acid) 1:3:0.75 (incorporation of stearic acid)	65 (incorporation of oleic acid) 60 (incorporation of stearic acid)	24	*w*(palmitic acid)=45.3%, *w*(palmitic acid at *sn*-2 position)=70%	(*15*)
Lipozyme RM IM	tripalmitin and a mixture of hazelnut oil fatty acids, EPA and DHA	hexane	batch	10	1:12.4	55	24	*w*(palmitic acid at *sn*-2 position)=76.6%, *w*(EPA+DHA)_total_=5%, *w*(oleic acid)=40%	(*46*)
Lipozyme RM IM	lard and a mixture of FFAs from palm kernel oil, tea seed oil and soybean oil	solvent-free system	batch	7	1:2	60	1	fatty acid composition resembles HMF	(*48*)
Lipozyme RM IM	tripalmitin and a mixture of hazelnut oil fatty acids and MCFAs	hexane	batch	19.78	1:3.35	57	24	*w*(caprylic acid)=12.8%, *w*(capric acid)=10.6%, *w*(palmitic acid)=30%	(*41*)
Lipozyme RM IM	palm stearin and mixed of stearic acid, myristic acid, and FFAs from rapeseed oil, sunflower oil and palm kernel oil	solvent-free system	batch	10.7	1:14.6	57	3.4	*w*(palmitic acid)=29.7%, *w*(palmitic acid at *sn*-2 position)=62.8%	(*52*)
Lipozyme RM IM	palm stearin and rapeseed oil fatty acids	solvent-free system	batch	8	1:10	60	4	*w*(palmitic acid at *sn*-2 position)=83.7%	(*49*)
Lipozyme RM IM	palm stearin and mixed of stearic acid, myristic acid, and FFAs from rapeseed oil, sunflower oil and palm kernel oil	solvent-free system	packed bed reactor	-	1:9.5	58	2.7	*w*(palmitic acid)=28.8%, *w*(palmitic acid at *sn*-2 position)=53.2%	(*53*)
Lipozyme RM IM	34L‐leaf lard and camellia oil fatty acids	solvent-free system	batch	6	1:4	45	6	*w*(oleic acid)=51.5%, *w*(palmitic acid at *sn*-2 position)=84%	(*45*)
Lipozyme RM IM	tripalmitin and FFAs from silkworm pupae oil	hexane	batch	10	1:12	65	48	*w*(palmitic acid at *sn*-2 position)=97.05%	(*44*)
Lipozyme RM IM	tripalmitin and PUFAs from microalgal oil	hexane	batch	7	1:7	60	21	*w*(palmitic acid at *sn*-2 position)=89.0%, *w*(PUFAs at *sn*-1,3 position)=81.3%	(*40*)
Lipozyme RM IM	solid fraction of basa catfish oil and high oleic sunflower oil fatty acids	solvent-free system	batch	12	1:6	50	2	*w*(palmitic acid at *sn*-2 position)=57.8%, *w*(oleic acid at *sn*-1,3 position)=79.21%	(*50*)
Lipozyme RM IM	basa catfish oil and sesame fatty acids	solvent-free system	batch	8	1:3	40	2	*w*(palmitic acid)=23.8%, *w*(palmitic acid at *sn*-2 position)= 48.3%	(*51*)
Lipozyme RM IM	mixture of palm stearin fractions and fungal oil from *Mortierella alpina* and oleic acid	solvent-free system	batch	8	1:6	60	6	*w*(fatty acids at *sn*-2 position)/%: palmitic acid 68.7%, ARA 9.8%, oleic acid 7.9%	(*47*)
Lipozyme RM IM	palm stearin fractions, oleic acid and linoleic acid	solvent-free system	batch	8	1:8:4	60	4	*w*(OPO and OPL)=69.26%, *w*(palmitic acid at *sn*-2)=87.75%	(*75*)
Lipozyme TL IM	tripalmitin, extra virgin olive oil fatty acids and DHA	solvent-free system	batch	10	1:3:2, 1:4:2 and 1:5:1	65	24	*x*(palmitic acid at *sn*-2 position)= 60%, *x*(DHA)_total_=7.5% (1:3:2), 6.72% (1:4:2), 5.89% (1:5:1)	(*76*)
Lipozyme TL IM	tripalmitin and a mixture of FFAs from hazelnut oil and *Echium* oil	hexane	batch	10	1:4	60	8	*w*(SDA)= 2.0%, *x*(oleic acid)= 22.9%, *x*(palmitic acid at *sn*-2 position)= 46.2%	(*62*)
Lipzyme IM-20	tripalmitin and oleic acid	isooctane	batch	8.33	1:5	40	12	*w*(OPO)= 55.2%	(*81*)
Novozyme 435	palm olein, DHA and ARA	hexane	batch	10	1:18	60	24	*γ*(DHA+ARA incorporated at *sn*-2 position)=17.20 g/100 g	(*67*)
Novozyme 435	palm olein and a mixture of 23.23% DHA, 31.42% GLA and 15.12% palmitic acid	hexane	batch	10	1:2	60	22.7	*w*(palmitic acid at *sn*-2 position)= 35.11%, *w*(DHA)= 3.75%, *w*(GLA)= 5.03%	(*68*)
*Candida* sp. 99-125	lard and oleic acid	solvent-free system	batch	10	1:2	45	10	*w*(OPO)=55.3% (using additive β-cyclodextrin)	(*32*)
Lipozyme RM IM and Lipozyme TL IM	tripalmitin and a mixture of hazelnut fatty acids and GLA	hexane	batch	10 (Lipozyme RM IM) 6 (Lipozyme TL IM)	1:14.8 (Lipozyme RM IM) 1:14 (Lipozyme TL IM)	55	24	*w*(palmitic acid at *sn*-2 position)=74.9% (Lipozyme RM IM), 73.9% (Lipozyme TL IM)	(*16*)
Lipozyme RM IM, Lipozyme TL IM, Novozyme 435 and *Candida parapsilosis*	system I: 3.9 g tripalmitin and 2.76 g oleic acid system II: 3.9 g tripalmitin and 3.17 g ω-3 PUFA	solvent-free system	batch	8.9 (from tripalmitin)	1:2	60	24	*w*(palmitic acid at *sn*-2 position)=79.9% (Lipozyme RM IM), 75.6% (Lipozyme TL IM), 61.2% (Novozyme 435), 87.3% (*Candida parapsilosis*)	(*60*)
Novozyme 435, recombinant CAL-B, Lipozyme TL IM and Lipozyme RM IM	TAG from *Nannochloropsis oculata* and *Isochrysis galbana* fatty acids	solvent-free system	batch	10	1:3	60 (Novozyme 435, Lipozyme TL IM) 50 (recombinant CAL-B, Lipozyme RM IM)	24	*w*(ω-3 PUFAs)_total_=13.92–17.12% *w*(palmitic acid at *sn*-2 position)= 59.38–68.13%	(*6*)

FFAs=free fatty acids, PUFAS=polyunsaturated fatty acids, MCFAs=medium-chain fatty acids, EPA=eicosapentaenoic acid, GLA=γ-linolenic acid, DHA=docosahexaenoic acid, CAL-B=*Candida antartica* lipase B, ARA=arachidonic acid, TAG=triacylglycerol, OPO=1,3-dioleoyl-2-palmitoylglycerol, OPL=1-oleoyl-2-palmitoyl-3-linoleoylglycerol, SDA=stearidonic acid

Transesterification is a reaction between TAG and TAG or ester (*4*). HMFS-based transesterification reaction is carried out with TAGs that are rich in palmitic acid at the *sn*-2 position, using *sn*-1,3 regioselective lipase. In the transesterification reaction, the final product is a mixture of different TAGs with similar physical properties. To some extent, it is difficult to purify and obtain the target HMFS. Nevertheless, this issue is specific, depending on the type and proportion of oil (*5*, *9*). Table 4 (*43*, *55*-*59*, *66*, *69*, *86*-*92*) summarizes the reaction conditions for HMFS synthesis by transesterification.

**Table 4:** Process conditions for the production of human milk fat substitute through transesterification

Lipase	Substrate	Solvent system	Operation mode	Enzyme loading/%	Substrate ratio	Temperature/°C	Time/h	Product	Reference
Lipozyme TL IM	palm oil, palm kernel oil, olive oil, sunflower oil and marine oil	solvent-free system	batch	10	*ζ*=4.0:3.5:1.0:1.5:0.2	60	6	*w*(palmitic acid at *sn*-2 position)=41.5%	(*58*)
Lipozyme TL IM	palm stearin fractions and ethyl oleate	solvent-free system	batch	10	*r*=1:5.5	50	3	*w*(OPO)=31.43%, *w*(palmitic acid at *sn*-2 position)=80.6%	(*59*)
Lipozyme TL IM	tripalmitin and mixed of extra virgin olive oil and flaxseed oil	solvent free system	batch	10	*r*=1:2.67	56.7	18	*w*(palmitic acid)=25.2%, *w*(α-linolenic acid)=15.9%	(*57*)
Lipozyme TL IM	palm stearin fractions and fish oil	solvent-free system	batch	10	*r*=2:1	60	12	*w*(palmitic acid at *sn*-2 position)=75.98%	(*56*)
Lipozyme RM IM	tripalmitin and ARA ethyl ester from fungi *Mortierella alpina*	hexane	batch	7.58	*r*=1:12	50	13.36	*w*(ARA at *sn*-1,3 positions)=69.57%, *w*(palmitic acid at *sn*-2 position)=62.31%	(*43*)
Novozyme 435	hazelnut oil and ethyl palmitate	hexane and solvent-free system	batch	10	*r*=1:6	65	17	*w*(palmitic acid at *sn*-2 position)=35.5% (mg scale, hexane) and 71.1% (g scale, solvent-free system)	(*66*)
Novozyme 435	palm stearin, palm kernel oil, soybean oil, olive oil and tuna fish oil	solvent-free system	batch	10	*ζ*=2.9:3.4:1.5:2.0:0.2	60	4	fatty acid composition resembling HMF	(*69*)
*Candida parapsilosis*	tripalmitin and ethyl oleate	solvent-free system	batch	5 (from tripalmitin)	*r*=1:8	60	24	*w*(oleic acid at *sn*-2 position)=15%	(*86*)
recombinant CAL-B	fungal oil from *Mortierella alpina* and MCT	solvent-free system	batch	8	*r*=1:1	90	3	*w*(MMM)=6.12%, *w*(MLCT)=53.75%, *w*(LLL)=40.13%	(*87*)
Lipozyme RM IM	basa catfish oil and coconut oil	solvent-free system	batch	8	*r*=1.5:1	60	3	*w*(MLCT)=62.14%, *w*(MLL)=39.85%, *w*(palmitic acid at *sn*-2 position)=46.14%	(*55*)
Lipozyme RM IM	lard and milk thistle oil	solvent-free system	batch	8	*ζ*=8:2	60	4	*w*(palmitic acid)=21% and about 75% palmitic acid at *sn*-2 position	(*88*)
Lipozyme RM IM	lard and milk thistle oil	solvent-free system	batch	8	*ζ*=8:2	70	4	*w*(palmitic acid at *sn*-2 position)>70%	(*89*)
Lipozyme RM IM	lard and rapeseed oil	solvent-free system	batch	8	*ζ*=8:2	70	4	*w*(palmitic acid at *sn*-2 position)=41.6%	(*90*)
Lipozyme RM IM	lard, sunflower oil, canola oil, palm kernel oil, palm oil, algae oil and microbial oil	solvent-free system	batch	11	*ζ*=1.00:0.10:0.50:0.13:0.12:0.02:0.02	60	3	*w*(palmitic acid)=20.1% and *w*(palmitic acid at *sn*-2 position)=38.2%	(*91*)
Lipozyme RM IM	lard, sunflower oil, canola oil, palm kernel oil, palm oil, algae oil and microbial oil	solvent-free system	packed bed reactor	-	*ζ*=1.00:0.10:0.50:0.13:0.12:0.02:0.02	50	1.5	*w*(palmitic acid at *sn*-2 position)=39.2%	(*92*)

MMM=medium-medium-medium chain fatty acids, MLL=medium-long-long chain fatty acids, LLL=long-long-long chain fatty acids, MLCT=medium-long chain triacylglycerol, OPO=1,3-dioleoyl-2-palmitoylglycerol, ARA=arachidonic acid, HMF=human milk fat

The one-step enzymatic process has been used in many studies due to its simplicity, but its drawbacks are: (*i*) difficulties in converting intermediate DAGs into desired HMFS resembling HMF, and (*ii*) complexity of purification due to the presence of by-products (*24*). To overcome these drawbacks, a multi-step enzymatic process such as alcoholysis followed by esterification has been proposed (*9*, *24*). The synthesis of HMFS *via* multi-step enzymatic process results in a higher OPO purity (74-95%) than in one-step enzymatic process (about 43%). However, this approach also has bottlenecks, especially the reaction complexity and high solvent consumption (*45*).

The two-step process for the HMFS synthesis can be alcoholysis route followed by the esterification reaction (*42*, *93*, *94*) (Table 5). The two-step process is proposed in the synthesis of HMFS to overcome the drawbacks of acidolysis and transesterification. This method exploits the regioselectivity of lipase at *sn*-1,3 (*9*, *24*). Two-step synthesis consists of alcoholysis of TAG using *sn*-1,3 specific lipase to produce *sn*-2 MAG rich in palmitic acid and followed by esterification of *sn*-2 MAG rich in palmitic acid with FFAs (*4*, *95*) or esterified fatty acids (*93*). Generally, the final product of interesterification between *sn*-2 MAG rich in palmitic acid and oleic acid contains 92-94% palmitic acid at *sn*-2 position and 83-89% oleic acid at *sn*-1,3 position, while the yield of OPO reaches 70-72% (*9*). The alcoholysis followed by esterification process avoids the acyl migration and obtains a purely structured TAG (HMFS) (*5*, *9*, *24*). However, this process is not commonly used in industrial production due to the complexity of the steps, which leads to an increased overall cost (*9*).

**Table 5:** Process conditions for human milk fat substitute production through two-step reactions (two-step acidolysis, alcoholysis-esterification)

Lipase	Substrate	Solvent system	Operation mode	Enzyme loading/%	Substrate ratio	Temperature/°C	Time/h	Products	Reference
*Rhizopus oryzae*, *Mucor miehei*, Lipozyme RM IM, Lipozyme TL IM and *Alcaligenes* sp.	TAG containing high palmitic acid at *sn*-2 position (acidolysis of palm stearin and palmitic acid) and oleic acid	hexane and solvent-free system	batch stirred tank reactor	13.3 (hexane) 3.3 (solvent-free system)	*r*=1:6	37 (hexane) 50 (solvent-free systme)	1 (hexane) 4 or 19 (solvent-free system)	*w*(palmitic acid at *sn*-2 position)=67.8% and 66.0% in hexane and solvent-free system, respectively	(*61*)
acidolysis I: Novozyme 435 acidolysis II: *Rhizopus oryzae* immobilized in Accurel MP-1000	acidolysis I: tuna fish oil and palmitic acid acidolysis II: neutralized product acidolysis I and oleic acid	hexane	batch stirred tank reactor	acidolysis I: 10 acidolysis II: 13.3	acidolysis I: *ζ*=1:1 acidolysis II: *r*=1:6	37	acidolysis I: 48 acidolysis II: 1	acidolysis I: *r*(palmitic acid at *sn*-2 position)=5% Acidolysis II: *r*(palmitic acid)=52%, *r*(DHA)=15%, *r*(linoleic acid at *sn*-2 position)=3%	(*65*)
Lipozyme RM IM	2-monoglyceride from alcoholysis of palm stearin, then esterified with methyl ester from coconut oil	hexane	batch	10	*ζ*=1:3	50	12	*w*(MCFAs)=43.86%, *w*(lauric acid)=39.37%, *w*(palmitic acid at *sn*-2 position)=24.18%	(*93*)
Lipozyme RM IM	2-palmitoylmonoacylglycerol from ethanolysis of lard using Novozyme 435, oleic acid, linoleic acid and lard	hexane	batch	10	*r*=3:5.2:3.5:7	37	6	fatty acid composition resembles HMF	(*42*)
*Candida* sp. 99-125	monopalmitin at *sn*-2 positon from alcoholysis of tripalmitin, then esterified with oleic acid	solvent-free system	batch	9	*r*=4.5:18	38	1.5	*Υ*(OPO)=65%, *w*(palmitic acid at *sn*-2 position)=75%	(*94*)

TAG=triacylglycerol, DHA=docosahexaenoic acid, MCFAs=medium-chain fatty acids, OPO=1,3-dioleoyl-2-palmitoylglycerol

Two-step synthesis of HMFS can also be carried out through two-step acidolysis (*61*, *65*) (Table 4). Esteban *et al.* (*61*) conducted acidolysis of palm stearin and palmitic acid at *r*=1:3, temperature 37 °C in solvent system using Novozyme 435, which produced TAGs containing a high palmitic acid content at the *sn*-2 position (74.5%). After the first acidolysis, the obtained TAGs were used as intermediates for the second acidolysis with oleic acid at *r*=1:6 using *R. oryzae*, *Mucor miehei*, RM IM, TL IM and *Alcaligenes* sp. lipases. The final product contained 67.8% palmitic acid at *sn*-2 position and 67.2% oleic acid at *sn*-1,3 positions (*61*). In addition, Pina-Rodriguez and Akoh (*96*) carried out a two-step interesterification (transesterification followed by acidolysis) for the synthesis of the DHA-containing amaranth oil structured lipid. First, a customized amaranth oil was produced by transesterification of amaranth oil and ethyl palmitate using Novozyme 435. The second step was acidolysis of the obtained oil with DHA using Lipozyme RM IM. The final product contained 28% palmitic acid and 33% palmitic acid at *sn*-2 position.

The interesterification for HMFS synthesis can be carried out in batch and continuous reactors (*5*). The batch reactor is easy to operate and suitable for small scale production. However, at the industrial scale, for an economical production process, continuous operation is preferred rather than batch-wise operation (*53*, *97*). In continuous reactor system, such as continuous stirred tank reactor (CSTR), plug flow reactor (PFR) or packed bed reactor (PBR), substrate is continuously introduced into the reactor and the product is subsequently withdrawn (*98*). PBR is more suitable for industrial-scale production than CSTR (*24*).

The advantages of PBR over the batch reactor for the production of structured lipids can be seen in the following aspects (*53*): (*i*) the slow substrate flow through the enzyme column to avoid the damage of the enzyme structure and increase the stability of the enzyme, (*ii*) the production can be carried out continuously, and (*iii*) it reduces the occurrence of acyl migration due to the excessive use of the enzyme. To some extent, the continuous process at a high volumetric flow rate is more advantageous than operation at a slow volumetric flow rate. At a high flow rate, possibility of acyl migration reduces, thus increasing the productivity (*23*). The acyl migration in a PBR is lower than in a stirred batch reactor (*24*). Nielsen *et al.* (*97*) reported that the reaction equilibrium in acidolysis of lard and soybean oil fatty acids in a PBR was reached in <1.5 h residence time. Zou *et al.* (*53*) reported that Lipozyme RM IM could be used for 10 days in a PBR without a significant loss of activity in interesterification between palm stearin and mixed of stearic acid, myristic acid and fatty acids from rapeseed oil, sunflower oil and palm kernel oil. Wang *et al.* (*40*) also reported that the number of reuses of lipase in a packed reactor increased 2.25-fold compared to that of batch reactor.

## FACTORS INFLUENCING HMFS SYNTHESIS

Some aspects considered for HMFS synthesis are biocatalyst concentration, reaction type, substrate composition and mode of operation (*5*). Table 3, Table 4 and Table 5 show selected works on HMFS synthesis using various substrates, enzymes and other relevant parameters for optimizing the process in order to obtain products that resemble HMF.

### Effect of lipase concentration

Lipase concentration affects the rate of interesterification reaction. The initial reaction rate increases by the increase of lipase concentration due to a higher number of active side pockets available for catalytic activities (*71*, *93*, *94*). Lipase concentration also affects the amount of DAG and the rate of acyl migration (*51*). A higher lipase concentration enhances the incorporation of the acyl donors (acyl migration) in acidolysis (*39*). Some published reports that are shown in Table 3, Table 4 and Table 5 are not comparable because the related reaction conditions are not provided (*i.e.* the enzyme activity and the amount of substrate). It is worth mentioning that lipase concentration must be optimized. To some extent, the progressive increase of lipase concentration promotes the synthesis of OPO *via* shortening the reaction time and weakening the acyl migration (*99*). However, the excessive enzyme amount will favour hydrolytic reaction over esterification.

Zou *et al.* (*51*) reported that after reaction time of 2 h in acidolysis between basa catfish oil and fatty acids from sesame oil using 2% Lipozyme RM IM, the content of *sn*-2 palmitate was 56%. At enzyme loading of 8, 11 and 14%, the amounts of *sn*-2 palmitate were about 63.5-65%. Ilyasoglu *et al.* (*57*) used 10% Lipozyme TL IM in transesterification between tripalmitin and a mixture of extra virgin olive oil with flaxseed oil to produce HMFS with 25.2% palmitic acid and 15.9% α-linolenic acid. Jiménez *et al.* (*37*) used 10% Novozyme 435 in acidolysis between palm stearin and palmitic acid to produce TAGs containing 70.5% palmitic acid at *sn*-2 position. In general, the range of enzyme loads for HMFS synthesis by interesterification is 8-10%.

### Effect of moisture content

The enzyme inactivation due to dehydration sometimes causes poor interesterification (*33*). Lipase has high activity in the nearly absent-to-micro-aqueous system, typically interface-activated at the oil-water interface (*71*). The hydrolysis is usually considered the rate-limiting reaction in which water acts as a reactant. To some extent, the enhancement of moisture content increases the initial activity of lipase. However, excessive water entails the formation of by-products (*39*). A small amount of water is important for lipase to maintain its activation (*i.e.* lubrication of the enzyme conformation). Therefore, the amount of water must be controlled especially during acidolysis (*51*).

Zheng *et al.* (*71*) reported that OPO content reached a maximum conversion (43.9%) at 2% moisture content during the interesterification of tripalmitin and oleic acid. This conversion decreased as the moisture content increased. In other studies, the addition of 1% moisture content in acidolysis of lard and oleic acid increased OPO yield from 52.8 to 55.3%, whereas at 5% moisture content, the OPO content gradually decreased (*32*). Zou *et al.* (*51*) reported the optimum moisture content in acidolysis between palm stearin and FFAs for HMFS synthesis of about 0.24%. Thus, the range of water content in HMFS synthesis by enzymatic interesterification is 0.2-2%.

### Effect of solvent

Generally, lipase-catalyzed interesterification for HMFS synthesis can be performed in either solvent system (*i.e.* organic solvents) or solvent-free system. The solvent increases the solubility of high-melting-point reactants. Thus, the reaction can be operated at a lower temperature, which is beneficial for the enzyme stability. However, excessive solvent amount dilutes the reaction fluid and reduces the random access of substrate to the lipase active sites (*94*). Several factors must be considered when selecting a proper solvent for a particular enzymatic reaction including: (*i*) compatibility of the solvent with the reaction, (*ii*) solvent properties (density, viscosity, surface tension, toxicity, flammability), and (*iii*) cost. Lipase tends to be more active in *n*-hexane than in other solvents such as isooctane, acetone, petroleum ether, toluene, or ethyl acetate. *n*-Hexane plays a key role in increasing the solubility of non-polar substrates and shifting the reaction towards esterification rather than hydrolysis (*24*).

Palmitic acid-enriched TAG has a high melting point so it requires a higher temperature in the solvent-free reaction system in order to keep the substrate liquid during the reaction (*61*). Palm stearin and palmitic acid have high melting points so they are difficult to react without a solvent as they require a minimum temperature of 65 °C (*37*). Esteban *et al.* (*61*) reported that the incorporation of oleic acid at the *sn*-1,3 position was slightly lower in the solvent-free system (46.2%) than in the solvent system (50.4%) in the interesterification between palmitic acid-enriched TAG from palm stearin and oleic acid. It was caused by a lower reaction rate due to a lower mass transfer rate when no solvent is available. In addition, Cao *et al.* (*100*) reported that in acidolysis, the rate of acyl migration and the concentration of intermediate or side products (*e.g*. DAG and MAG) decreased significantly in the anhydrous reaction system.

### Effect of substrate ratio

The interesterification reaction rate of HMFS synthesis depends on the amount of substrate ratio (TAG to acyl donor) after the reaction equilibrium has been achieved (*39*, *71*). Enhancement of the amount of substance ratio of TAG to fatty acids leads to a reaction equilibrium (*32*, *39*, *101*), and produces the desired incorporation of fatty acids into TAG (*6*). The presence of excessive TAG substrate reduces lipase active site capabilities. Also, an excessive FFA amount causes environmental acidification, increases the viscosity of the system, inhibits biocatalyst activity and reduces mass transfer rate (*71*). The high amount of TAG to fatty acid ratio may increase the frequency of collisions between the enzyme and substrates (*102*). The increase of palmitic acid content at *sn*-2 position is greater when the amount of substance ratio of TAG to fatty acid is enhanced in the interesterification between palmitic acid-enriched TAG from palm stearin and oleic acid (*61*). The amount of substrate ratio also affects fatty acids at the *sn*-1,3 positions. Increasing the amount of substrate ratio decreased the saturated fatty acid content at *sn*-1,3 position in acidolysis between a mixture of palm stearin and ARA oil with oleic acid (*47*).

Bryś *et al*. (*88*) reported transesterification between lard and milk thistle oil at a mass ratio 6:4 and 8:2 at 60 °C using 8% Lipozyme RM IM. After 4 h at the amount of substrate ratio 8:2, HMFS with 21% palmitic acid and about 75% palmitic acid at the *sn*-2 position was obtained. Meanwhile, at a ratio of 6:4, HMFS contained less than 70% palmitic acid at the *sn*-2 position. In addition, Tecelão *et al.* (*86*) reported that the incorporation of oleic acid increased drastically (from *r*=32 to 51%) by raising the substrate ratio of tripalmitin to ethyl oleate from 1:2 to 1:8. Zou *et al.* (*52*) reported that at the optimum amount of substrate ratio for acidolysis between palm stearin and a mixture of stearic acid, myristic acid and FFAs from rapeseed oil, sunflower oil and palm kernel oil of 1:14.6 yielded HMFS with 29.7% palmitic acid and 62.8% palmitic acid at *sn*-2 position. Generally, the range of the amount of substrate ratio (*i.e.* tripalmitin, palm stearin, lard and catfish oil) to fatty acids in the interesterification for HMFS synthesis is from 1:2 to 1:14.

### Effect of reaction temperature

The reaction temperature influences the subtle variations in the architecture/conformation of lipase and leads to thermal inactivation of lipase and reduction in the affinity between the substrate and the biocatalyst (*103*). A higher temperature enhances the mass transfer and, to some extent, increases the activity of lipase as well (*94*). In endothermic reactions, higher temperatures provide better results due to the shift in thermodynamic balance. At high temperatures, the operation of the process is also easy as the solubility of the reactants increases and the viscosity of the solution decreases (*39*). Moderately high temperatures can provide sufficient energy to overcome the reaction barrier, while too high temperatures can cause lipase thermal deactivation (*104*). Therefore, the reaction temperature should be considered as low as possible so that the reaction efficiency and product quality are ensured (*51*). The optimal temperature will vary with different lipase sources (*6*, *105*). The reaction temperature is positively correlated with acyl migration. It also has an effect on the acyl incorporation (*106*) in which high temperatures may facilitate acyl migration (*39*).

The OPO content reached a maximum (46.5%) at a reaction temperature of 50 °C for the interesterification between tripalmitin and oleic acid using CLL@mMWCNTs. However, the OPO content decreased with the increase in reaction temperatures, especially above 50 °C (*71*). He *et al.* (*6*) reported that the highest amount of ω-3 PUFAs (13.92-17.12%) in HMFS was obtained by interesterification between TAG from *Nannochloropsis oculata* and fatty acids from *Isochrysis galbana* using Novozyme 435, recombinant CAL-B lipase, Lipozyme TL IM and Lipozyme RM IM at reaction temperatures of 60, 50, 60 and 50 °C, respectively. Generally, the range of reaction temperatures for HMFS synthesis *via* enzymatic interesterification is 40-60 °C.

### Effect of reaction time

The reaction yield for the synthesis of structured lipids is positively affected by an increase in reaction time (*57*, *66*). Reaction time in the interesterification is governed by the reactor configuration (*i.e.* batch or continuous reactor) (*40*). Wang *et al.* (*40*) reported that the reaction time for HMFS synthesis *via* interesterification between tripalmitin and PUFAs from microalgal oil in PBR (2.5 h) was faster than that of batch reactor (7 h). Generally, the reaction time in a batch reactor is the factor that the most affects the increase in acyl migration and eventually results in the production of the partial acylglycerols such as DAG and MAG. The acyl migration increases linearly with an increased reaction time (*59*). In addition, the reaction temperature also affects the reaction time. Yang *et al.* (*39*) reported the interesterification between lard and fatty acids from soybean where the reaction time to reach incorporation of 20% linoleic acid and 3% linolenic acid decreased with the increase of reaction temperature from 5 h at 50 °C down to 2.4 h at 90 °C.

Bryś *et al*. (*89*) reported that transesterification between lard and milk thistle oil at a mass ratio 8:2 using 8% Lipozyme RM IM at 70 °C yielded HMFS with above 70% palmitic acid at the *sn*-2 position after 2 and 6 h, but only 53.4% after 4 h. In addition, Bryś *et al*. (*90*) also reported transesterification between lard and rapeseed oil at a mass ratio 8:2 using 8% Lipozyme RM IM at 70 °C for 4 h. The produced HMFS had 24.2% palmitic acid and 41.6% palmitic acid at the *sn*-2 position. On the other hand, after 8 and 24 h of reaction, HMFS had 34.9 and 26.4% palmitic acid at the *sn*-2 position, respectively. The OPO content in the product of interesterification between tripalmitin-rich palm stearin and ethyl oleate in a batch process using Lipozyme TL IM decreased from 29.3 to 18.5% as the reaction time increased from 3 to 12 h, respectively (*59*). In addition, Zou *et al.* (*53*) reported the interesterification between palm stearin and a mixture of stearic, myristic and fatty acids from rapeseed, sunflower and palm kernel oil, respectively, in PBR with the following reaction conditions: residence time 2.7 h, temperature 58 °C and amount of substrate ratio 1:9.5. Under these conditions, the contents of palmitic acid in TAGs and at *sn*-2 position were 28.8 and 53.2%, respectively. Generally, the range of reaction time for HMFS synthesis *via* enzymatic interesterification in a batch process is 2-24 h, while in a continuous process it is 1-3 h.

## PURIFICATION OF HMFS

The synthesis of structured lipids by the enzymatic interesterification produces TAGs, partial glycerides (DAG and MAG) and FFAs. The acidolysis between TAG and fatty acids gives products with a high FFA content. Products of acidolysis between palm stearin and palmitic acid at an amount of substance ratio 1:3 contain 50% FFAs (*37*). The transesterification between TAG molecules gives products with low content of FFAs (0.5-7%) (*56*, *58*, *69*). Thus, each type of enzymatic interesterification or utilization of different substrates can result in different complexity in the purification of HMFS. This complexity, as indicated earlier, depends on the number of by-products contained in the reaction mixture. Purification after HMFS synthesis is intended to increase TAG fraction by removing FFAs and partial glycerides. The removal of FFAs can be carried out by neutralization (*57*, *61*, *62*, *65*, *82*), liquid-liquid extraction (*55*, *83*) and evaporation using molecular distillation (*45*, *48*, *50*, *51*, *53*, *84*, *85*). Molecular distillation is also applied to remove both FFAs and partial glycerides simultaneously (*48*).

Neutralization is carried out through saponification of FFAs using an alkaline solution such as KOH. The acylglycerol fraction is then extracted using hexane (*57*, *61*, *62*, *65*, *82*). Ilyasoglu (*57*) reported that the neutralization of the transesterification product of tripalmitin and a mixture of olive oil and flaxseed oil (1:1) (*r*=1:2.67) using 0.8 M KOH enhanced TAG content up to 78%. Robles *et al*. (*65*) also reported the neutralization of the acidolysis product of palm stearin rich in palmitic acid at the *sn*-2 position and oleic acid (*r*=1:6) using 0.5 M KOH at 37 °C. The TAG yield was up to 80%. Esteban *et al*. (*61*) confirmed the neutralization of acidolysis product of palm stearin rich in palmitic acid at the *sn*-2 position and oleic acid using 0.5 M KOH in the presence or absence of hexane. With (at room temperature) and without solvent at 50 °C, the neutralization can increase TAG purity to 99% with the yield of 96%. Yuan *et al*. (*55*) reported the removal of FFAs from the interesterification product using liquid-liquid extraction with 85% ethanol at a volume ratio of 1:1.

Separation using molecular distillation is carried out based on the difference in vaporization temperatures of FFAs, partial glycerides and TAGs. Using molecular distillation, Qin *et al*. (*45*) purified the acidolysis product of 34L-leaf lard and camellia fatty acids (*r*=1:4). At the evaporation temperature of 180 °C and pressure of 6.7-7.5 Pa, the TAGs were rich in OPO with the purity of 91.39% and the yield of 40.75%. Zou *et al*. (*50*) also reported the purification of the product of acidolysis between the solid fraction of basa catfish oil and high oleic sunflower oil fatty acids (*r*=1:6). At the evaporation temperature of 185 °C and the pressure of 2 Pa, the TAG fraction with the yield of 95.7% was obtained. A stepwise evaporation using molecular distillation is also possible for purification of interesterification product. Sørensen *et al*. (*84*) produced TAG fraction of 31.3% from the acidolysis between butterfat and a mixture of fatty acids from rapeseed oil and soybean oil (*r*=1:2). The conditions were pressure of 0.1 Pa and the evaporation temperatures in stages 1 and 2 of 90 and 185 °C, respectively. The ranges of evaporation temperatures and pressures of molecular distillation to remove FFAs during HMFS purification are 180-185 °C and 0.1-7.5 Pa. In addition, the separation of TAGs from partial glycerides is carried out at the evaporation temperature of 230 °C and a pressure of 10^7^ Pa (*48*).

In the two-step acidolysis (*i.e.* a multi-stage process), purification starts with the first acidolysis to remove FFAs from the reaction mixture. In the second acidolysis, FFAs and DAGs are also removed from the product mixture. A single-step enzymatic process can also produce nearly pure HMFS. However, it is challenging to convert all of the intermediate DAGs formed during the reaction. In addition, multiple purification steps are required to remove the by-products (*24*). The concentration of target TAGs containing palmitic acid at the *sn*-2 position in the final product can be increased by separating the other TAGs through fractionated crystallization (*58*, *81*, *84*). Lee *et al*. (*81*) reported an increased OPO content from 25.2 to 53.3% in the enzymatic transesterification product of palm oil and camellia oil by fractionation at 22 °C for 16 h. Sørensen *et al*. (*84*) reported that HMFS with 56% palmitic acid at the *sn*-2 position was produced from fractionation of the acidolysis product of butterfat and a mixture of fatty acids from rapeseed and soybean oil. Also, the acidolysis product of solid fractions from fractionation of butterfat and a mixture of fatty acids from rapeseed and soybean oil produced HMFS with 47% palmitic acid at the *sn*-2 position.

## CURRENT DEVELOPMENT OF HMFS PRODUCTION

In the last two decades, HMFS has been developed from a wide variety of substrates and enzymes and under various reaction conditions. In general, the most studied type of HMFS is *sn*-2 palmitate (OPO) because this TAG is the major component of HMF. Thus, the main consideration in HMFS production is to have palmitic acid at the *sn*-2 position (*107*). OPO-enriched HMFS is produced from interesterification between palmitic acid-containing source (*i.e.* lard, tripalmitin, palm oil and its derivatives: palm stearin or palm olein, catfish oil, palmitic acid or ethyl palmitate) and oleic acid-containing sources (*i.e.* olive oil, high oleic sunflower oil, oleic acid or ethyl oleate).

A better understanding of the composition and structure of HMF leads to better HMFS investigations (*9*). Recently, Wang *et al.* (*75*) synthesized both OPL and OPO from palm stearin fractions. OPL synthesis has not received much attention. The OPO to OPL ratios in HMF range from 0.5 to 2.0 (*108*, *109*). Apart from *sn*-2 palmitate, HMF also contains PUFAs and MCFAs, which play an important role during the early human development (*4*, *110*).

Synthesis of HMFS enriched with long-chain polyunsaturated fatty acids can be obtained from fish oil, algal oil, fungal oil, microbial oil, silkworm pupae oil, hazelnut oil, soybean oil, sunflower oil, ALA, GLA, DHA and ARA. Ghosh *et al.* (*56*) synthesized HMFS from palm stearin fractions and fish oil (*r*=2:1) *via* transesterification using Lipozyme TL IM. The final product contained 75.98% palmitic acid at the *sn*-2 position, 0.27% ARA, 3.43% EPA and 4.25% DHA. Interesterification between tripalmitin and ARA ethyl ester from ARA-rich single-cell oil from *Mortierella alpina* (*r*=1:2) using Lipozyme RM IM resulted in 69.57% ARA incorporation at *sn*-1,3 position and 62.31% palmitic acid at *sn*-2 position (*43*).

Naturally, MCFAs are present in HMF in the form of medium- and long-chain triacylglycerols (MLCTs). The main composition of TAGs is one MCFA and two long-chain fatty acids (MLL type) (*4*, *111*). MCFAs in HMF mostly consist of lauric acid (C12:0), a small amount of capric acid (C10:0) and caprylic acid (C8:0) (*112*). MCT-enriched HMFS can also be synthesized from coconut oil, palm kernel oil and MCFAs containing caprylic acid and capric acid. Recently, Yuan *et al.* (*55*) synthesized HMFS containing TAG with MLL type from catfish oil and coconut oil (*r*=1.5:1) *via* interesterification using Lipozyme RM IM, Lipozyme TL IM, NS 40086 and DF Amano 15 (DF 15) with an enzyme load of 8%, temperature of 60 °C and reaction time of 3 h. The final product contained MLCT and MLL reaching 62.14 and 39.85%, respectively, with the main TAGs in HMFS being C12:0/C16:0/C18:1, C12:0/C18:1/C18:1, C12:0/C14:0/C18:1 and C12:0/C12:0/C18:1. Korma *et al.* (*87*) also produced HMFS containing 6.12% medium-medium-medium chain TAGs, 53.75% MLCTs and 40.13% long-long-long chain TAGs from interesterification of fungal oil containing high ARA content from *Mortierella alpina* and MCT (*r*=1:1) catalyzed by 8% recombinant CAL-B, temperature of 90 °C and reaction time of 3 h.

A single-step enzymatic transesterification can produce HMFS similar to HMF using the suited amount of substrate ratio. For example, Zou *et al*. (*91*, *92*) reported a mixture of lard, sunflower oil, canola oil, palm kernel oil, palm oil, algal oil and microbial oil at a mass ratio 1.00:0.10:0.50:0.13:0.12:0.02:0.02 for HMFS synthesis. This substrate in the mixture was transesterified at a temperature of 60 °C, moisture content of 3.5% (on the lipase mass basis), reaction time of 3 h and Lipozyme RM IM 11% (on the total substrate mass basis). The product of HMFS had palmitic acid content of 20.1% with 38.2% palmitic acid at *sn*-2 position. The resulting HMFS had a high degree of similarity with HMF in the composition of total and *sn*-2 position fatty acids, PUFA and TAG with the values ​​of 92.5, 90.3, 61.5 and 71.9, respectively (*91*). Zou *et al*. (*92*) also used the substrate at that mixture ratio, which was transesterified using Lipozyme RM IM in PBR at 50 °C and a residence time of 1.5 h. The obtained HMFS had 39.2% palmitic acid at *sn*-2 position, 0.5% ARA and 0.3% DHA. Based on TAG content and purity, the degree of similarity of HMFS to HMF was 72.3.

At present, the commercial HMFS for inclusion in infant formulas has been successfully produced from various sources of oils and fats (*4*, *5*, *9*). The *sn*-2 palmitate is one of the structured TAGs that is generally supplemented into infant formulas (*5*, *113*). HMFS products that have been commercialized according to the main fatty acid composition are Betapol 60 (C16:0, C18:1, C18:2), InFat™ (C16:0, C18:1), Bonamil (C16:0, C18:1, C18:2, C18:3), Similac Advance (C16:0, C18:1, DHA, ARA), Alsoy (C16:0, C18:1, DHA, ARA), Aptamil 1 (C16:0, C18:1, EPA, DHA), Milu-Milk (C16:0, C18:1, DHA), Bledina Alma (C16:0, C18:1, C18:2), Gallia HA (C16:0, C18:1, DHA, ARA), Cow & Gate Premium (C16:0, C18:1, DHA, GLA, ARA) and Baby Semp 1 (C16:0, C18:1, DHA, ARA) (*24*).

## OUTLOOK: CHALLENGES AND OPPORTUNITIES IN HMFS SYNTHESIS

Structured lipids are designed through the modification of oils and fats to have desired nutritional or physicochemical properties suitable for food industry (*9*, *114*, *115*). HMFS is one of the ingredients in infant formula that is potentially and continuously developed to support infant growth according to the needs of each stage of baby’s age (*i.e.* infant and advanced formulas) and baby conditions (normal or premature and low birth mass babies).

The challenge for developing HMFS is the relatively high production cost. To enhance productivity (thus, reducing overall production cost), the synthesis of HMFS is carried out through a careful selection of the substrate, enzyme, reactor configuration and reaction conditions. Generally, the optimum reaction conditions for HMFS synthesis are at amount of substrate ratios between TAGs and FFAs of 1:2-1:14, temperatures of 40-60 °C, enzyme loads of 8-10% and reaction times of 2-24 h in batch process or 1-3 h in continuous process. Large-scale production of HMFS through a one-stage process using tripalmitin is not attractive because of its high cost and difficulty in obtaining products resembling HMF (*24*). On the other hand, the multistep reaction can produce higher yield of HMFS that has properties resembling HMF. However, the increase in reaction system complexity will also tend to increase downstreaming costs. It is worth mentioning that the production of HMFS in a solvent-free system is preferred in terms of food safety and costs (*5*).

One of the potential sources of substrates for HMFS synthesis is palm stearin because of its high palmitic acid content and relatively low price. However, the content of palmitic acid-rich TAGs at the *sn*-2 position of palm stearin needs to be increased through chemical interesterification (*52*, *53*), enzymatic interesterification (*37*, *38*) or fractionation (*47*, *56*, *59*, *116*), which is due to the nature of palm stearin that is abundant in oleic acid at the *sn*-2 position. The acidolysis between palm stearin and oleic acid using an *sn*-1,3-specific lipase will result in triolein, which is not preferred (*75*). The HMFS synthetic route using palm stearin has to be started with enhancement of palmitic acid-rich TAGs at the *sn*-2 position (*116*). Then, the fatty acids at the *sn*-1,3 positions from the palmitic acid-rich TAGs are replaced with acyl donors through acidolysis or transesterification. The common acyl donors are single fatty acids (oleic acid, ALA, GLA, EPA, DHA and ARA), FFA mixtures of vegetable oils (such as olive, camelina, rapeseed, sunflower or hazelnut oil), sources of ω-3 PUFAs (such as fish or microalgal oil) (*5*, *9*, *24*), or sources of MCFA (such as coconut or palm kernel oil). HMFS that is similar to HMF and has C8:0, C10:0, C12:0, C16:0, C18:1, C18:2, EPA, DHA, GLA and ARA can potentially be commercialized in the future.

In HMFS synthesis, the high ratio of acyl donors is not attractive due to the difficulties in the separation process (such as deacidification) (*51*). This entails high costs of post-process separation (*32*). The possibility of producing HMFS with a low ratio of acyl donors is very interesting. However, the main limitation in the reaction process is low mass transfer, thus, a lower reaction rate. To overcome this problem, an enzyme that has a higher specificity and stability is needed. Faustino *et al.* (*77*) reported that tripalmitin consumption of 62.7% was achieved at *r*=1:1.2 at 65 °C using *R. oryzae* lipase immobilized on Lewatit VPOC 1600 during acidolysis between tripalmitin and FFAs from camelina oil. The isolation and genetic engineering of new lipases with better stability during operation at high temperatures are also of interest for future research (*9*, *19*). The mutagenesis techniques are also promising for creating novel lipases such as an *sn*-2-specific lipase (*22*), which would facilitate the production of OPO. In addition, the use of continuous systems other than PBR, such as enzymatic membrane reactor, is also interesting to be developed (*117*). In enzymatic membrane reactor system, a continuous reaction can be facilitated by having immobilized enzyme retained inside the reactor (*117*).

## CONCLUSIONS

Human milk fat substitute (HMFS) is synthesized by the enzymatic interesterification of vegetable oils, animal fats or blends of oils. The main characteristic of HMFS is having triacylglycerols (TAGs) with palmitic acid located at the *sn*-2 position and unsaturated fatty acids at the *sn*-1,3 positions. Selection of substrates, enzymes, batch or continuous reactor configuration and reaction conditions needs to be considered to increase the overall production of HMFS. Lipozyme RM IM, Lipozyme TL IM and Novozyme 435 are widely used for the synthesis of HMFS. Lipozyme RM IM and Lipozyme TL IM are used as biocatalysts due to their regiospecificity towards *sn*-1,3 positions. Generally, Lipozyme RM IM is used in acidolysis, whereas Lipozyme TL IM is used in transesterification. Novozyme 435 is used due to its regiospecificity towards *sn*-2 position, which is beneficial for incorporating palmitic acid at *sn*-2 position of the oils and fats, both in acidolysis and transesterification. Generally, the optimum reaction conditions for HMFS synthesis are amount of substrate ratios of TAGs and fatty acids between 1:2 and 1:14, temperatures of 40-60 °C, enzyme loads of 8-10%, moisture contents of 0.2-2% and reaction times of 2-24 h in batch process or 1-3 h in continuous process. The separation of interesterification product from FFAs in HMFS synthesis is carried out by neutralization using 0.5 M KOH (1.5 times the quantity of KOH required to neutralize the FFAs) or molecular distillation at the evaporation temperatures of 180-185 °C and pressures of 0.1-7.5 Pa.

117SitanggangABDrewsAKraumeM. Development of a continuous membrane reactor process for enzyme-catalyzed lactulose synthesis. Biochem Eng J. 2016;109:65–80. 10.1016/j.bej.2016.01.006
